# A Coregulatory Network of NR2F1 and microRNA-140

**DOI:** 10.1371/journal.pone.0083358

**Published:** 2013-12-03

**Authors:** David Y. Chiang, David W. Cuthbertson, Fernanda R. Ruiz, Na Li, Fred A. Pereira

**Affiliations:** 1 Department of Molecular Physiology and Biophysics, Baylor College of Medicine, Houston, Texas, United States of America; 2 Interdepartmental Program in Translational Biology and Molecular Medicine, Baylor College of Medicine, Houston, Texas, United States of America; 3 Bobby R. Alford Department of Otolaryngology- Head and Neck Surgery, Baylor College of Medicine, Houston, Texas, United States of America; 4 Huffington Center on Aging and Department of Molecular and Cellular Biology, Baylor College of Medicine, Houston, Texas, United States of America; Texas A&M University, United States of America

## Abstract

**Background:**

Both nuclear receptor subfamily 2 group F member 1 (NR2F1) and microRNAs (miRNAs) have been shown to play critical roles in the developing and functional inner ear. Based on previous studies suggesting interplay between NR2F1 and miRNAs, we investigated the coregulation between NR2F1 and miRNAs to better understand the regulatory mechanisms of inner ear development and functional maturation.

**Results:**

Using a bioinformatic approach, we identified 11 potential miRNAs that might coregulate target genes with NR2F1 and analyzed their targets and potential roles in physiology and disease. We selected 6 miRNAs to analyze using quantitative real-time (qRT) -PCR and found that miR-140 is significantly down-regulated by 4.5-fold (P=0.004) in the inner ear of NR2F1 knockout (*Nr2f1^–/–^*) mice compared to wild-type littermates but is unchanged in the brain. Based on this, we performed chromatin-immunoprecipitation followed by qRT-PCR and confirmed that NR2F1 directly binds and regulates both *miR-140* and *Klf9* *in* *vivo*. Furthermore, we performed luciferase reporter assay and showed that miR-140 mimic directly regulates *KLF9-3’UTR*, thereby establishing and validating an example coregulatory network involving NR2F1, miR-140, and *Klf9*.

**Conclusions:**

We have described and experimentally validated a novel tissue-dependent coregulatory network for NR2F1, miR-140, and *Klf9* in the inner ear and we propose the existence of many such coregulatory networks important for both inner ear development and function.

## Introduction

Nuclear receptors are a large family of cellular regulators that function as activators, repressors and silencers of transcription when bound by specific ligands or cofactors [1,2]. They are structured with a ligand-binding domain in the C-terminus, activation function domains, and a DNA-binding domain in the N-terminus which binds to specific hormone response DNA elements in the genome [3]. Two major subclasses of nuclear receptors include the nuclear hormone receptors that respond to hormone ligands and the orphan nuclear receptors for which endogenous ligands are not known [4]. Nuclear receptor subfamily 2 group F member 1 (NR2F1) is an orphan nuclear receptor required for the development of the inner ear and cerebral cortex [5–8]. This is illustrated by loss-of-function in knockout mice (*Nr2f1* ^*–/–*^) which impacts neurogenesis, axonal guidance, neocortical patterning, morphogenesis, and patterning of the cochlear sensory epithelium and the inner and outer hair cells in the organ of Corti [7–10]. A recent forward genetics screen in patients with congenital anomalies and cytogenetically balanced chromosomal rearrangements identified a critical paracentric microdeletion of the *Nr2f1* locus, implicating haploinsufficiency of NR2F1 as a cause for a 4-year-old child’s deafness, dysmorphism and developmental delay [11].

In an effort to define transcription factor binding sites and target genes *in vivo*, Montemayor et al. developed a methodology to identify NR2F1 target genes by intersecting gene expression profiling, computational binding site queries, and evolutionary conservation data [12]. This approach is in part based on the concept of ‘phylogenetic footprinting,’ which assumes evolution selects against mutations within DNA regions that have an important regulatory function; thus creating evolutionary *cold spots* within the critical genomic segments that regulate gene expression [13]. Using this approach we identified and validated several direct target genes of NR2F1, and began to uncover regulatory and feedback networks at the transcriptional and post-translational level that might be necessary in the development and physiology of mammals. To date, relatively few coregulatory networks have been delineated for interactions between nuclear receptors and microRNAs (miRNAs), and even fewer have been experimentally validated [14]. 

MicroRNAs were discovered as small temporal RNAs that regulate developmental transitions in *C. elegans*. They have diverse expressive patterns and regulate a variety of areas, including embryologic development, physiology, pathophysiology, and growth, development, progression, and metastasis in cancer [15–17]. Currently, there are 2,578 (human) and 1908 (mouse) mature unique miRNAs identified (mirBase 20, June 2013), and more than half are conserved in vertebrates [18]. In humans, genes encoding miRNAs are located in intergenic (52%), intragenic–intronic (43%), or intragenic-exonic (5%) regions with both sense and antisense orientations [19]. This large family of 21-23 nucleotide single-stranded non-coding RNAs negatively regulate gene expression at the post-transcriptional level by binding the 5’UTR, coding sequences, or 3’UTR. They have the ability to either promote degradation or suppress the translation of their target mRNA, depending on the complementarity with which they bind [15,16]. These small non-coding RNAs are first formed as pri-miRNAs which are capped, adenylated, spliced, and later cleaved by Drosha and Pasha endonucleases (RNase-III type) resulting in precursor miRNAs (pre-miRNA) [16]. Pre-miRNAs are then processed by Dicer to form short duplex RNAs, which are incorporated into a miRNA-induced silencing complex (miRISC), in order to interact with the target mRNA. 

MicroRNAs have a specific role in the developing inner ear and hearing, where many miRNA expression profiles change both temporally and spatially during embryogenesis and post-natal maturation and are conserved through evolution [20–22]. The significance of miRNAs in audition is exemplified by mutations in the seed region of human miRNA-96 causing a nonsyndromic progressive hearing loss in humans [21]. Furthermore, *Dicer* is required for survival of inner ear hair cells in mice, and a loss-of-function results in aberrant hair bundle formation, stereocilia defects, disrupted inner ear morphogenesis, and impaired hearing function [23,24].

Given the apparent roles of NR2F1 and miRNAs in the ontogenesis of the inner ear, and more notably the functional deficits caused when these regulators are mutated or deleted, we sought to discover the genetic coregulatory networks of NR2F1 and miRNAs. We hypothesized that knowing if, how, and when gene regulatory pathways intersect could shed light on the current incomplete knowledge of the regulatory mechanisms of inner ear development and functional maturation [20], and perhaps extend understanding to other organ systems as well. Such mechanistic understanding is a crucial step in discovering innovative ways of protecting, regenerating or rejuvenating hair cells and neurons in patients with partial or complete hearing loss [25,26]. This study discovered coregulatory interactions between NR2F1 and miRNA-140 in their regulation of the gene Krüppel-like factor 9 (*Klf9*). This is the first description of such a pathway and delineates the regulatory actions of a circuit involving the non-steroidal nuclear receptor transcription factor and miRNAs.

## Materials and Methods

### Ethics Statement

The Institutional Animal Care and Use Committee of Baylor College of Medicine approved all studies involving the use of animals as mandated by the United States federal government. 

### Identification of the 11 miRNAs and their putative targets

In order to find the overlap of any miRNA gene with NR2F1 target genes, the human genome (GRCh37/hg19) and mouse genome (NCBI37/mm9) builds were viewed using the UCSC genome browser [27] (http://genome.ucsc.edu) with the NR2F1 binding site custom track supplied by Montemayor et al. [12]. The miRNA track was also viewed to identify every miRNA mentioned by Wang et al. to be expressed in the developing mouse inner ear [22]. Other miRNAs were identified by reviewing the literature involving ontogenesis or NR2F1[21,22,28–30]. To find all possible gene targets for each miRNA, we utilized multiple database prediction tools: miRBase (http://www.mirbase.org) [31], MicroCosm (http://www.ebi.ac.uk/enright-srv/microcosm/htdocs/targets/v5), TargetScan 6.0 (http://www.targetscan.org), microRNA.org (http://www.microrna.org/microrna/home .do), and PITA (http://genie.weizmann.ac.il/pubs/mir07/mir07_dyn_data.html) [32]. The predicted genes from the different databases were combined and redundant genes removed. The final lists were compared with the putative NR2F1 targets identified by Montemayor et al. [12].

### miRNA binding site predictions

MicroRNA.org (http://www.microrna.org/microrna/home .do) was used to determine binding sites within the translated regions of the genes while TargetScan 6.2 (http://www.targetscan.org) [33] and miRWalk (http://www.umm.uni-heidelberg.de/apps/zmf/mirwalk/index.html) [34] were used to determine binding sites in the 3’ UTR. The range of the binding sites was manually curated. 

### Functional annotation and biological significance

The Database for Annotation, Visualization and Integrated Discovery (DAVID) [35,36] querying UniProt and the Genetic Association Database was used to determine the expression pattern and associated neurological diseases of the 28 genes targeted by at least 3 of the 11 miRNAs. Functional clusters were discovered using the Gene Functional Classification function in DAVID. Cancer-related genes targeted by both NR2F1 and the 11 miRNAs were identified by searching the Sanger Institute’s cancer database. To discover genes that affect hearing loss, the hereditary hearing loss databases (hereditaryhearingloss.org), the Corey Lab at Harvard [37,38], and commercial hearing loss databases (SABiosciences, Frederick, MD) were mined and compared with the putative targets of NR2F1 and the 11 miRNAs. In addition, we examined genes involved in circadian rhythm have been shown to be involved in many aspects of embryogenesis [39] and identified 18 genes that are targeted by at least one of the 11 miRNAs. Finally, lists of genes involved in Notch signaling, neurogenesis, and stem cell function were obtained from SABiosciences (Frederick, MD) and filtered against the putative targets of the 11 miRNAs. 

### RNA extraction and real-time polymerase chain reaction

Inner ears of P0 *Nr2f1* ^*–/–*^ (–/–) and WT (+/+) littermates were placed in RNA Later (Ambion) after dissection, stored at 4°C overnight and then at -20°C until the genotype was determined [9]. Total RNA was isolated using the RNeasy Mini Kit (Qiagen) according to the manufacturer’s instructions and quantified using NanoDrop spectrophotometry (Wilmington, DE). 

Reverse transcription and real-time PCR of mRNAs were carried out using 0.25μg of RNA, nuclease-free water, 0.5μl of Oligo(dT), 10μl 5x First-Strand buffer, 4μl of 10mM dNTP, 5μl of 0.1M DTT, 1μl ribonuclease inhibitor (Invitrogen), and 2μl SuperScript II (Invitrogen). Reverse transcription was performed at 42°C for 1h before heating to 70°C for 15 min then returning to 4°C. Real-time PCR was performed in triplicates in 96-well plates in Mastercycler ep realplex (Eppendorf, Hamburg, Germany) using the following program: 1) 50°C for 2min, 2) 95°C for 2min, 3) 45 cycles of 95°C for 15s, 60°C for 1min, and 4) melting curve ramp. 

Reverse transcription and real-time PCR of microRNAs were carried out using a protocol modified from Varkonyi-Gasic et al. using stem-loop primers and SYBR Green without any commercial miRNA detection kit [40]. All stem-loop, forward and reverse primers were taken from Tang et al. [41] except for the stem-loop and forward primers for mmu-miR-1192 which have the sequences 5’-ctcaactggtgtcgtggagtcggcaattcagttgagaatttggt-3’ and 5’- acactccagctgggaaacaaacaaaca-3’. Briefly, 0.25μg of RNA for each sample was incubated for 5 minutes at 65°C with nuclease-free water and 0.5μl of 10mM dNTP mix in a final volume of 12.65μl and returned to ice for at least 2 minutes. A mix of 4μl 5X First-Strand buffer, 2μl 0.1M DTT, 0.1μl ribonuclease inhibitor (Invitrogen), 0.25μl SuperScript II RT (Invitrogen) and 1μl of 1μM stem-loop primer was added for each reaction. Reverse transcription was performed using the following program: 1) 16°C for 30min, 2) 45 cycles at 30°C for 30s, 42°C for 30s, and 50°C for 1s, and 3) 85°C for 5min. For real-time PCR, 2μl of the reverse transcribed samples were mixed with 1μl each of the forward and reverse primers (1μM), 4μl of Express SYBR Green (Invitrogen) and 12μl nuclease-free water. The real-time PCR was performed in triplicates in 96-well plates in Mastercycler ep realplex (Eppendorf, Hamburg, Germany) using the following program: 1) 95°C for 5min, 2) 45 cycles of 95°C for 5s, 60°C for 10s, 72°C for 1s, 3) melting curve ramp from 65°C to 95°C at 0.1°C per second. 

Expression levels of both mRNA and miRNAs were compared using the relative C_T_ (cycle number) method [42] after normalization to the expression level of cyclophilin A. Primer sequences are included in Table **S7** in File **S1**.

### Chromatin-immunoprecipitation

Cerebral cortex was harvested from C57Bl/6 mice and processed by chromatin crosslinking and immunoprecipitation as described in the Active Motif ChIP-IT Express Kit (Active Motif, Carlsbad, CA). Briefly, 150 mg of cortex was minced and cross-linked with 1% formaldehyde at room temperature with gentle shaking for 12 min. Glycine was added (final concentration of 125 mM) to quench the formaldehyde by incubating for 5 min with gentle shaking. The tissue pieces were then dounce homogenized (loose clearance, 2ml, K885301-002, Kontes, Fisher Scientific, Waltham, MA) in 2 ml of ice-cold PBS supplemented with 10 ul proteinase inhibitor cocktail (PIC, Active Motif, Carlsbad, CA) and 10 ul phenylmethylsulfonyl fluoride (PMSF, 100mM, Active Motif, Carlsbad, CA) to obtain a single-cell slurry which were pelleted at 660xg at 4°C for 5 min. Cells were resuspended in 2 ml Lysis Buffer supplemented with PIC and PMSF, incubated for 30 min on ice before being dounce-homogenized again (tight clearance, 2ml, K885301-002, Kontes, Fisher Scientific, Waltham, MA) to release the nuclei, which were then pelleted with centrifugation for 10 min at 660xg rpm at 4°C. Chromatin fragmentation was performed in shearing buffer using a 450 Branson Sonifier (50 bursts of 30 sec pulse with 30 sec OFF, 30% power output and 90% duty cycle) and the sheared chromatin was collected at 20,000xg for 15 min at 4°C, after which the supernatant was stored at -80°C. 

Chromatin-immunoprecipitation (ChIP) was performed at 4°C overnight with antibodies against NR2F1 (Perseus proteomics Inc., PP-H8132, Tokyo, Japan) and histone H3K9-Ac (Cell signaling, C5B11, Danvers MA) while control immunoprecipitations (IPs) were performed with mouse IgG (Active Motif, Carlsbad, CA). Twenty microliters of magnetic beads (Active Motif), 2 ug of antibody, and the chromatin samples were rotated overnight at 4°C in 200 ul volumes each. The next day, beads were washed twice with 800 ul ChIP Buffer 1 and four times with 800 ul ChIP Buffer 2. The beads were then resuspended in Elution Buffer and rotated for 15 min at room temperature. Fifty microliters of Reverse-Cross-linking Buffer were added and the supernatant transferred to a fresh tube. ChIP and input samples were denatured at 95°C for 15 min in a BioRad thermocycler before the proteins were digested with 2 μl Proteinase K (0.5ug/ul) for 1 hour at 37°C. Genomic DNA was isolated using Qiagen PCR Kit (Qiagen, Valencia, CA) and used for RT-PCR as described above.

### Luciferase reporter assay

A luciferase reporter construct containing a 3304-bp fragment of the *KLF9* 3’ untranslated region (UTR) and an empty luciferase reporter construct were purchased from SwitchGear Genomics (Menlo Park, CA; #S813897 and #S890005, respectively). Putative miR-140 binding sites were identical with *Klf9* 3’UTR. miR-140 mimic and a non-targeting scramble miR mimic were purchased from Life Technologies (Grand Island, NY) and used at a final concentration of 30 nM. Transfection and luciferase assay were carried out according to the manufacturer’s published protocol [43], using HEK293 cells and LipoD293 as the transfection reagent (SignaGen Laboratories, Rockville, MD). Twenty-four hours after transfection, cells were harvested and luciferase signal assayed using LightSwitch Luciferase Assay Reagents (SwitchGear Genomics) according to the manufacturer’s instructions. Luciferase signal was normalized to the total protein concentration of the lysates. Three independent transfection experiments were performed. Statistical analysis was performed using two-tailed Student’s t-test with α=0.05.

### Statistics

Data are presented as mean ±SEM. Statistical analysis was performed using two-tailed Student’s t-test with α=0.05. 

## Results

### Intersection of NR2F1 targets and miRNAs expressed in the mouse inner ear

A multi-step approach was used to discover miRNAs that might participate to coregulate NR2F1 target genes. A list of putative NR2F1 target genes identified by Montemayor et al. [12] was used to find positional overlap between NR2F1 target genes with gene loci encoding miRNAs using the UCSC genome browser. Of all the NR2F1 target genes inspected, only one miRNA gene intersection was found in the human genome–the *Klf9* gene, which contains the gene encoding miR-1192, was previously validated to be up-regulated and inner ear of *Nr2f1* ^*–/–*^ mice [12]. Beyond that, the most proximal miRNA genes found to intersect NR2F1 target genes were those for miR-693 and miR-1954, which were approximately 18kbps upstream and downstream from the *Yipf3* and *Ak1* genes, respectively. 

Next, we investigated recent articles that identified miRNAs in the developing embryonic mouse inner ear [22–24,30,44]. Of the miRNAs expressed, miR-17 and miR-341 have NR2F1 binding sites within their coding regions, miR-140, miR-191 and miR-199b have NR2F1 binding sites within the proximal promoter region of their loci, and miR-183 and miR-181b have NR2F1 binding sites 8 and 15 base pairs immediately upstream of the transcription start sites, respectively. The miR-183 family of miRNAs is expressed and required for maintenance and survival of hair cells [45,46]. There were many others that had binding sites more distant from the miRNA genes and were not investigated in this study. Additionally, miR-96, miR-140, and miR-194 were included for the following reasons. miR-96 is the only miRNA demonstrated to cause hearing loss when mutated [21,28]; miR-140 is highly and selectively expressed in hair cells during development and targets Jag1 and RARβ, both important for hair cell and inner ear development [22,30]; miR-194 targets Fgf10, which is essential for the development of the otic vesicle [27,47,48]. Finally, miR-33 was included because it and NR2F1 are involved with cholesterol metabolism which may participate in hair cell development and function [29,49–51]. With these criteria a set of 11 miRNAs (miR-17, miR-33, miR-96, miR-140, miR-181b, miR-183, miR-191, miR-194, miR-199b, miR-341, and miR-1192) were selected that might participate to coordinate with NR2F1 to regulate inner ear gene expression.

### Identification of NR2F1 gene targets co-regulated by the 11 miRNAs

**Figure 1:**
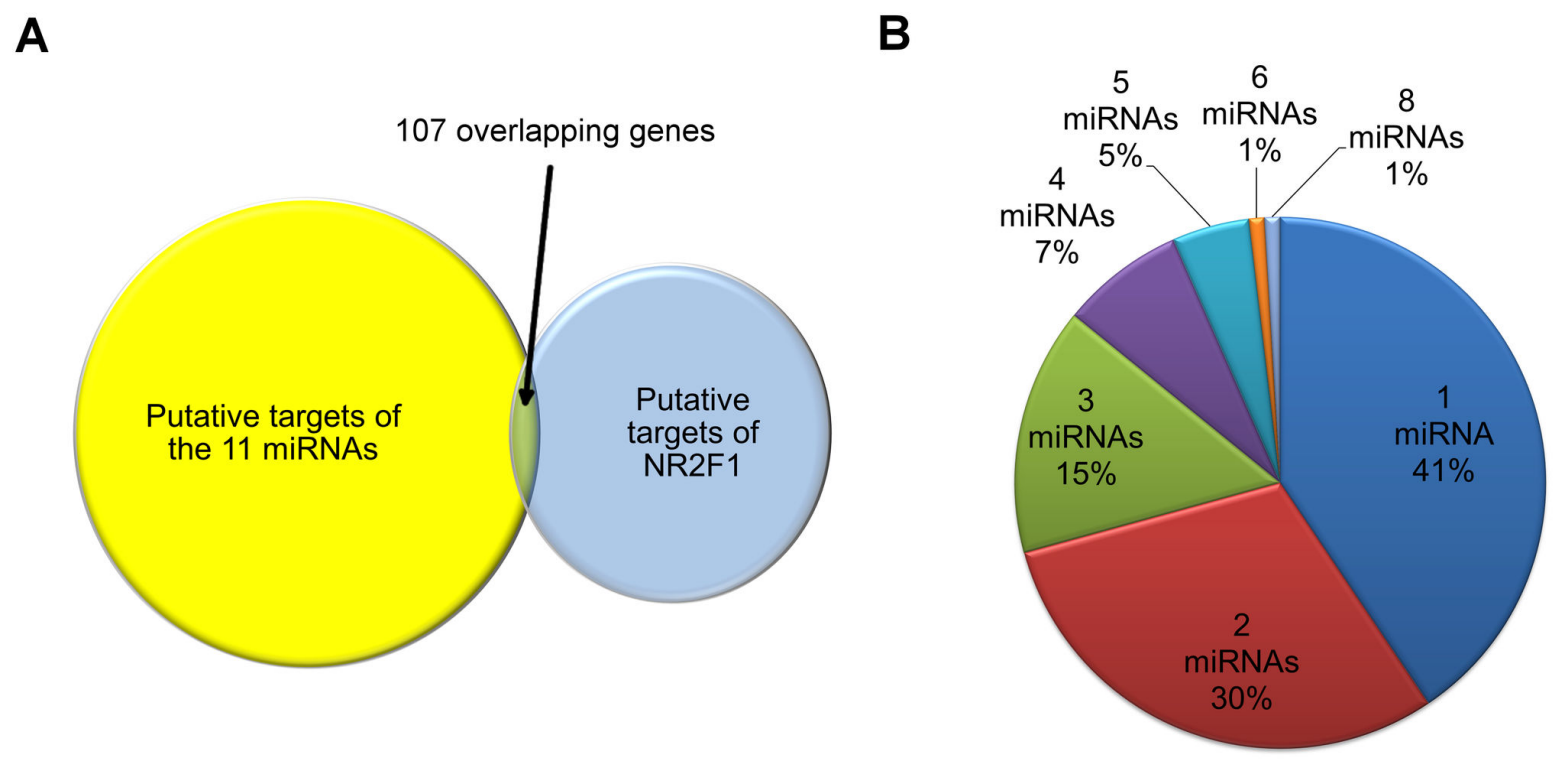
Intersection of genes targeted by both NR2F1 and select miRNAs. (A) Identification of 107 genes by intersection of the 182 NR2F1 targets genes [12] with targets of the 11 miRNAs and (B) the percentage of these genes targeted by multiple miRNAs.

To determine the relative potential for coregulation of target genes by NR2F1 and the miRNAs, we generated a list of the total number of genes targeted by the 11 miRNAs, as well as the subset of target genes whose expression was changed in the *Nr2f1* ^*–/–*^ mice, as quantified by microarray expression profiling and/or validated by qRT-PCR [12] (**Table S1 **in File **S1**). Of the 7201 unique miRNA genes targeted by the 11 miRNAs, 107 genes are also targeted by NR2F1 and 16 genes were previously validated to be changed in *Nr2f1* ^*–/–*^ [12] (Figure **1** and Table **1**). 

**Table 1:** List of 107 predicted miRNAs and NR2F1 coregulated inner ear targets.

MicroRNA	Putative miRNA/NR2F1 targets
mmu-miR17	*Acox1, Ahnak, Ampd3, C1qa, **Cidea**, Cldn1, Dlk1, Hsd17b11, Kcna5, **Klf9**, **Ldlr**, **Nr2f1**, Pdgfra, Ppia, Sfrs2, Sqstm1, Vps25*
mmu-miR-33	*Adora1, Ahnak, Ampd3, Ankrd1, **Casq1**, Ctbp1, Cxcl12, Ddx3x, Dusp1, Eif4b, **Fabp7**, Hspb8, Kcna5, Mmp14, Nefl, **Nr2f1**, Pdgfra, Sat1, Sfrs2, Txnrd1*
mmu-miR-96	*1810015C04Rik, Acan, Ahnak, Ak1, AK162128, Ampd3, Arg1, Cd83, Cldn1, Crym, Ddx3x, Elavl1, Foxk2, Glul, Hmgcs1, **Ldlr**, Man2a1, Myadm, Pdgfra, Psen1, **Sesn1**, Slc25a1, Snai2, **Stim1**, Tbx15, **Tectb**, Ttr, Tyms, Zrsr1*
mmu-miR-140	*Acox1, C1qc, Ddx3x, Dpep1, Eln, Gjb2, Gpx3, H2afy, **Klf9**, Man2a1, **Nr2f1**, Pdgfra, Prrx2, Ptgis, Rbp1, **Tectb**, Timm23, Trip4, Vpreb3, **Zbtb16***
mmu-miR-181b	*Acox1, Ahnak, Cd83, Chordc1, Crym, Cyr61, Ddit4, Ddx3x, Dr1, Eln, Erh, Foxk2, Hist1h2ao, Mmp14, Ms4a6b, Nbr1, Pdgfra, Pros1, Ran, Rbm8a, Sat1, Sfn, Snai2, Sqstm1, Tcn2*
mmu-miR-183	*2810410M20Rik, Acan, Ak1, Cldn1, Ddx3x, Dlk1, Eif4b, Foxk2, Gjb2, Ndufb6, Nefl, Ppia, Psen1, Ran, Sfrs2, Tbx15, Wdhd1*
mmu-miR-191	*Dr1, Elavl1, Foxk2, Mgp, Nbr1, Sat1, Satb1, Tsen34*
mmu-miR-194	*2410022L05Rik, Cd83, **Cidea**, Cldn1, Cxcl12, Cyp51, Dr1, Eln, Erh, **Fkbp5**, **Glul**, Hmgcs1, Nbr1, Pdgfra, Ran, Rasa4, Scg2, Sfrs2, Tmem106a, Txnrd1, Vps25, Ywhab*
mmu-miR-199b	*Aaas, C1qa, Ddit4, Dr1, Eln, **Fabp7**, **Ldlr**, Ltc4s, Pdgfra, Pros1, Psen1, Relb, Sat1, Sfrs2, **Stim1**, Tcp11, Tyro3, Vps4a*
mmu-miR-341	*C1qc, **Crabp1**, Hsd17b11, Jun, **Nfkbia**, Ptgis, Rbm8a*
mmu-miR-1192	*Ampd3, Chordc1, Dbi, Ddit4, Ddx3x, Dhx40, Dr1, Dusp1, Elavl1, **Fkbp5**, Foxk2, **Glul**, Hmgcs1, Jun, **Klf9**, **Ldlr**, Man2a1, **Nedd9**, Nefl, Pdgfra, Pros1, Ptgis, Ran, Satb1, Scg2, **Stim1**, Tbx15, Tek, Tsen34, Wdhd1, Ywhab*

Genes in bold type were previously validated to be changed in *Nr2f1* ^*–/–*^ tissues by qRT-PCR [12].

We next selected the genes targeted by at least 3 miRNAs for an in-depth analysis. This group of 28 genes, along with the miRNAs by which they are targeted, and the relative change in expression in the *Nr2f1* ^*–/–*^ [12] is shown in Table **S2** in File **S1**. All 28 genes are targeted by multiple miRNAs: 16 by 3 miRNAs, 8 by 4 miRNAs, 2 by 5 miRNAs, 1 each by 6 and 8 miRNAs. One notable miRNA target is *Nr2f1* itself that is targeted by three of these miRNAs (miR-17, -33 and -140), which suggests a feedback regulatory system may be possible. Other interesting observations include that *Pdgfrα*, a gene known for its role in both development and oncogenesis [52,53], was targeted by 8 of 11 miRNA, more than any of the other genes. Other genes with obvious roles in neurogenesis and neuron maintenance are also featured on this multi-hit list, such as *Psen1* and *Nefl* [54,55]. Analysis of the pathways, tissues, and diseases associated with each gene according to the Database for Annotation, Visualization and Integrated Discovery (DAVID) [35,36] querying the universal protein resource in UniProt [56] and the Genetic Association Database (GAD) [57], revealed 16 of the 28 genes (targeted by at least 3 miRNAs) are expressed in the human brain, and 18 of 24 genes have higher expression in the brain than other mouse tissues. Furthermore, several genes are known to cause diseases related to neurological development and are listed in Table **S3** in File **S1**. 

Multiple binding sites for a miRNA on a target mRNA might indicate greater regulatory control by that miRNA, whereas binding motifs shared by multiple miRNAs may indicate a lack of specificity in regulation [58]. Thus, we closely analyzed the specific miRNA binding site in both the untranslated (UTR) and translated regions of the target mRNA. In the 3’UTR, 14 of the target genes have only one motif per miRNA, 8 have two binding sites, and 6 have three or more binding sites and thus possibly maintain tighter regulatory control (**Table S4 **in File **S1**). In the translated portion of the mRNA of target genes, there were also some interesting finds as listed in Table **S5** in File **S1**. While there was no redundancy discovered in the 3’UTR of the target genes, 13 of the genes have binding motifs that are compatible with more than one miRNA. 

Regarding the genes that affect hearing loss, we mined databases from the Hereditary Hearing Loss (hereditaryhearingloss.org), the Serial Analysis of Gene Expression (SAGE) and the literature [37,38], and commercial hearing loss databases and found four genes to be targeted by both NR2F1 and miRNAs: *Crym* and *Snai2* were both targeted by miR-96 and miR-181b; *Gjb2* was targeted by miR-140 and miR-183 (**Table S5 **in File **S1**). In addition, 69 other genes implicated in hearing loss were targeted solely by the miRNAs but not NR2F1 (not shown).

### Functional clustering of the genes co-targeted by the 11 miRNAs and NR2F1

Using the Gene Ontology (GO) Gene Functional Classification analysis and the DAVID database [35,36], we discovered 9 functional clusters which includes all the 28 co-targeted genes (**Table S2 **in File **S1**) and contains 66 of the 107 genes targeted by at least 1 of the 11 miRNAs and NR2F1 (**Table S6 **in File **S1**). Based on GO enrichment score, Cluster 1 genes (*Crabp1, Fabp7, Rbp1*) involve cellular transport of fatty acids and hormones in metabolic process, including forebrain development, which is defective in *Nr2f1* ^*–/–*^. Cluster 2 genes (*Eif4b, Elavl1, Rbm8a, Sfrs2, Zrsr1*) involve RNA binding and metabolic processes and post-translational regulation of biological processes. The 10 genes in clusters 3-5 involve steroid, lipid and cholesterol biosynthesis, cation transport and chromatin assembly and organization, which are key processes during development. Cluster 6 has 20 genes that explicitly involve transcription, and include *Nr2f1* and *Klf9*. Clusters 7 through 9 have lower enrichment scores but involves skeletal development, nucleotide binding, cell adhesion, tube development and morphogenesis, the endomembrane system and energy regulation, many functions of which are defective in *Nr2f1* ^*–/–*^ [8,9,59].

### Validation of miRNA as targets using Nr2f1^–/–^ tissues

**Figure 2:**
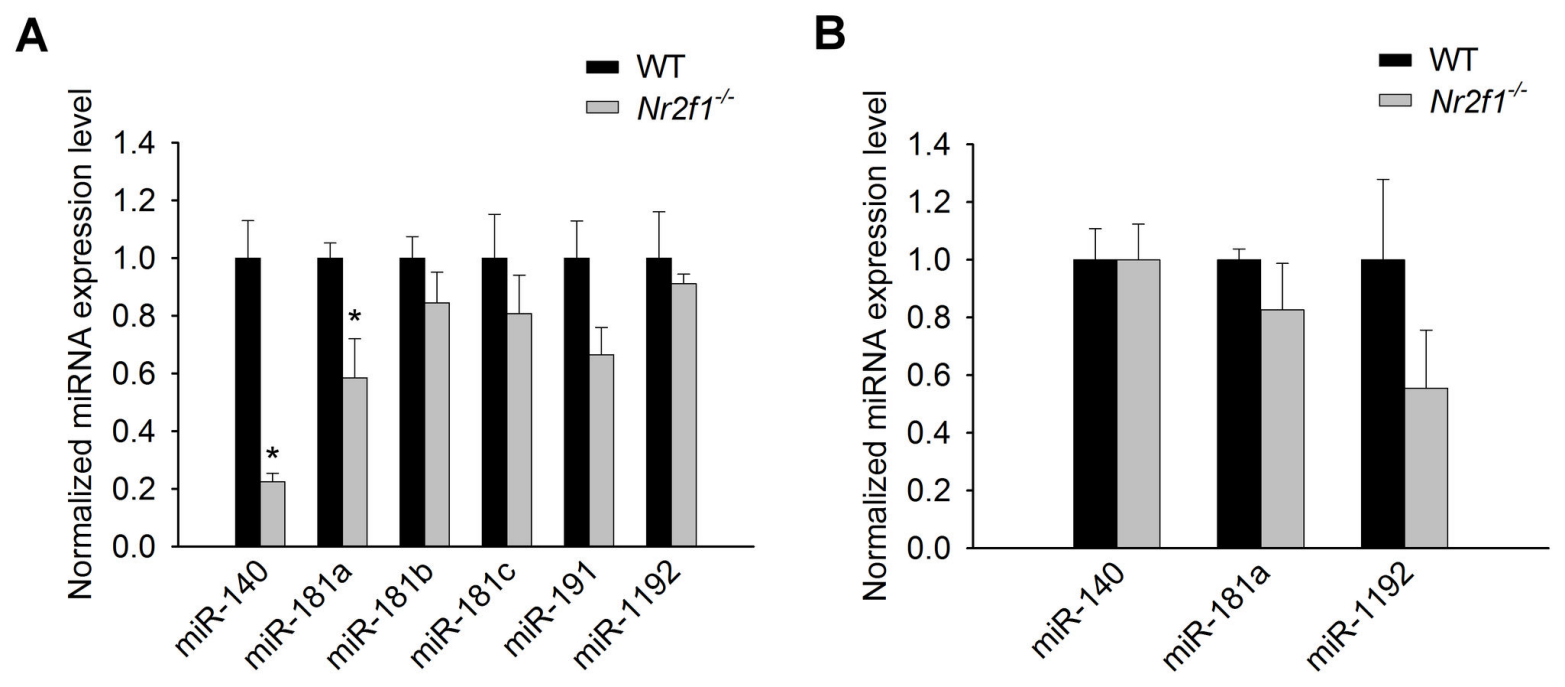
MicroRNA expression analyses in Nr2f1^–/–^ tissues. (A) The expression of select miRNAs in the Nr2f1^–/–^ inner ear showing miR-140 and miR-181a were significantly down-regulated by 4.5-fold (P=0.004) and 1.7-fold (P=0.046), respectively, compared to the WT. The levels of miR-181b, -181c and -191 were also decreased but did not reach significance (P=0.3, 0.4, 0.1, respectively). (B) The expression of miR-140 and miR-181a were unchanged in the cerebral cortex tissues from the same animals. N=3 in each group. Significance was assessed by a two-tailed t-test for independent comparison between Nr2f1^–/–^ and WT values for each miRNA with α=0.05.

In order to validate this bioinformatics approach, we pursued several miRNAs by determining their levels in the *Nr2f1* ^*–/–*^ mice using qRT-PCR. Of note, miR-140, -181b and -191 each have a putative NR2F1 binding site close to the proximal portion of their genes and since miR-181b is found in a family cluster we also included miR-181a and -181c, which may be coregulated and share similar gene targets. MicroRNA-140 has 4 co-targeted genes with NR2F1 amongst the validated expression changes in the *Nr2f1* ^*–/–*^ mice and is predicted to target NR2F1 itself (Table **1** & **S1** in File **S1**). miR-1192 is the only miR locus that is located within any of the NR2F1 target genes; it is found in the *Klf9* gene which is up-regulated in the inner ear of *Nr2f1* ^*–/–*^ mouse [12] and is targeted by miR-140 (Table **1**). By analyzing these 6 miRNAs using RT-PCR, we found that miR-140 and miR-181a are significantly down-regulated in the *Nr2f1* ^*–/–*^ inner ear by 4.5-fold (P=0.004) and 1.7-fold (P=0.046) compared with wildtype (WT), respectively, while the other 4 miRNAs were not significantly down-regulated (Figure **2A**). Because *Nr2f1* ^*–/–*^ mice have various neural defects [5,6,9–11], we went a step further to quantify the levels of miR-140, -181a, and -1192 in the cerebral cortex of the *Nr2f1* ^*–/–*^ but found that their levels were not significantly changed (Figure **2B**). This suggests a tissue-specific role for miR-140 and miR-181a in the *Nr2f1* ^*–/–*^ inner ear. 

### NR2F1 directly regulates miR-140

**Figure 3:**
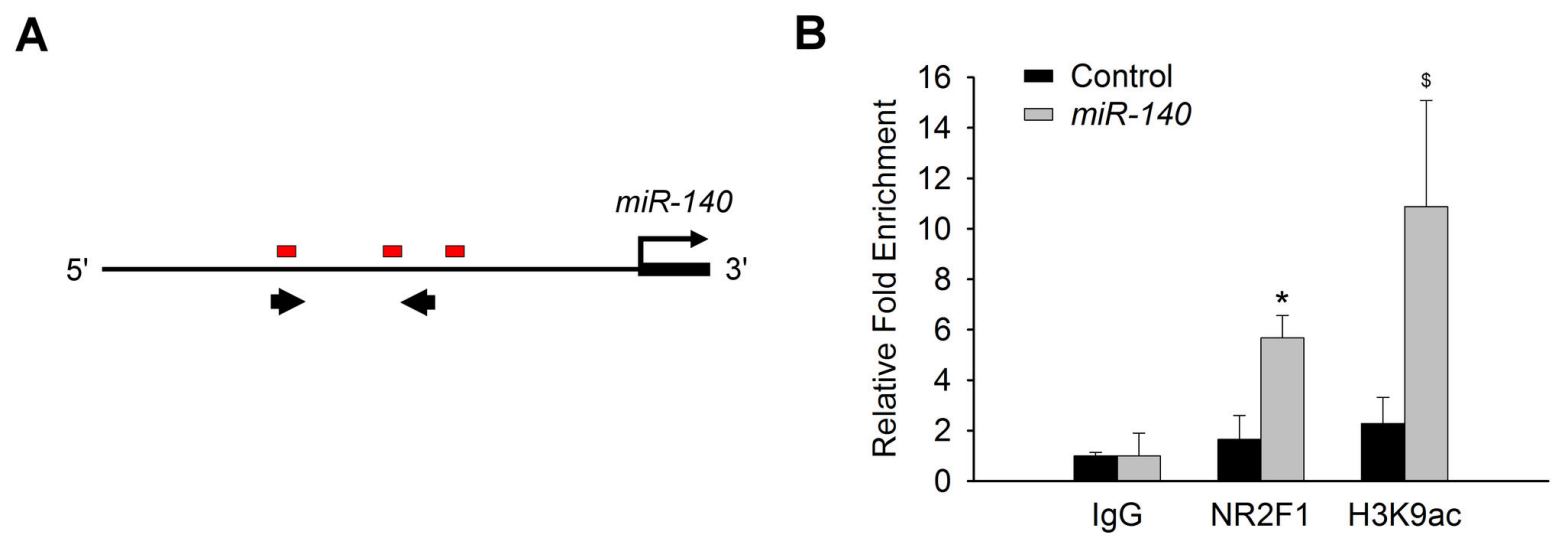
NR2F1 directly regulates *miR-140*. (A) Putative NR2F1 binding sites (red boxes) immediately upstream of the *miR-140* locus [12]. Thick arrows define regions of qRT-PCR amplification. (B) Chromatin-immunoprecipitation (ChIP) and qRT-PCR analyses reveal a significant enrichment of the *miR-140* locus with NR2F1 pull-down. N=5-6. *P<0.05, $P=0.1 vs IgG control.

Since the level of miR-140 was the most down-regulated in the absence of NR2F1 (Figure **2A**), we investigated whether NR2F1 might directly regulate miR-140 expression. We identified putative NR2F1 binding sites immediately upstream of the *miR-140* locus [12] (Figure **3A**) and performed chromatin-immunoprecipitation (ChIP) for NR2F1 binding enrichment followed by qRT-PCR analysis. Compared to a non-specific IgG control, there is a significant NR2F1 binding enrichment (P<0.05) at the *miR-140* locus (Figure **3B**) coincident with enrichment of the open chromatin marker acetylated H3K9, suggesting NR2F1 might directly regulate miR-140 transcription. 

### Tissue-Dependent Co-regulation of *Klf9* by NR2F1 and miR-140

**Figure 4:**
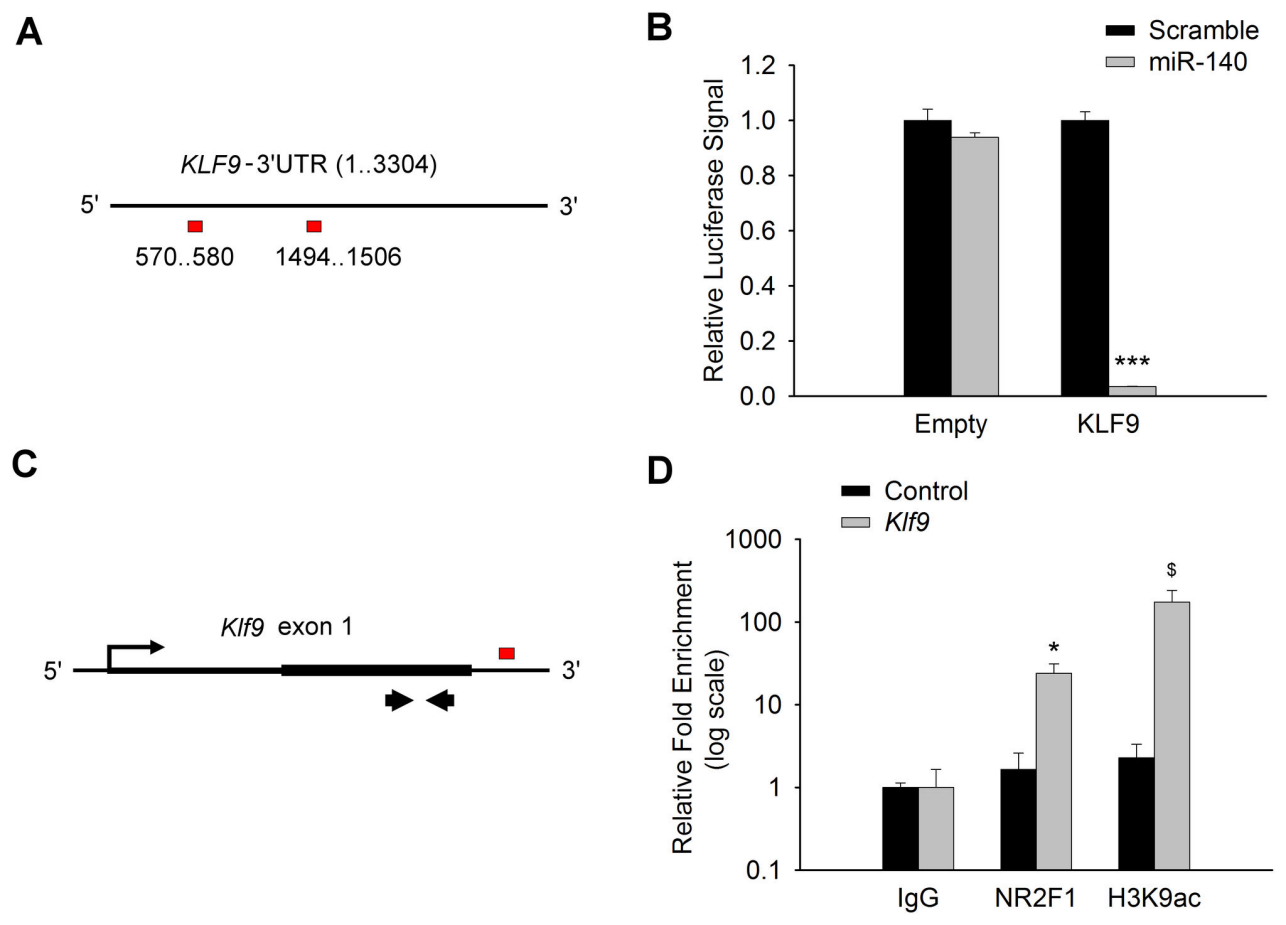
Both miR-140 and NR2F1 directly regulate Klf9 expression. (A) Putative miR-140 binding sites (red boxes) on *KLF9*-3’UTR. (B) Luciferase expression from a KLF9 3'UTR reporter is significantly reduced in the presence of the miR-140 mimic while Luc expression from the empty reporter is unchanged. N=3. ***P<0.001. (B) Schematic of the putative NR2F1 binding site (red box) just downstream of the first exon of Klf9 and the relative position of the qRT-PCR primers (Thick arrows). (D) Log-scale presentation of qRT-PCR results following chromatin-immunoprecipitation (ChIP) demonstrates significant enrichment of NR2F1 at the Klf9 locus. N=5. *P<0.05, $P=0.08 vs IgG control.

We found it intriguing that miR-1192 expression was not altered whereas the gene *Klf9*, which harbors the miR-1192 locus, was up-regulated in the *Nr2f1* ^*–/–*^ [12], suggesting that *Klf9* is regulated independently in the inner ear. *Klf9* is one of the 28 genes putatively targeted by both NR2F1 and at least 3 of the 11 miRNAs in this study (Figure **1** & Table **S2** in File **S1**). In particular, *Klf9* has multiple miR-140 binding sites: 3 sites in its 3’ UTR and 4 sites in its translated region (**Tables S4 & S5 **in File **S1**). To determine whether miR-140 regulates *Klf9* directly, we tested if a miR-140 mimic would target and affect the expression of a luciferase gene construct containing the *KLF9* 3’UTR sequences (Figure **4A**). We quantified Luciferase activity from the luciferase-*KLF9* 3'UTR reporter and found that miR-140 mimic reduced the Luciferase signal by more than 90% compared to a scrambled mimic (P<0.001, Figure **4B**). Next, we determined whether NR2F1 regulates *Klf9* directly by performing a ChIP followed by qRT-PCR using primers that amplify the *Klf9* first exon (Figure **4C**). Impressively, there was a 500-fold enrichment of NR2F1 binding at the *Klf9* locus as compared to a non-specific IgG control (P<0.05) (Figure **4D**). 

**Figure 5:**
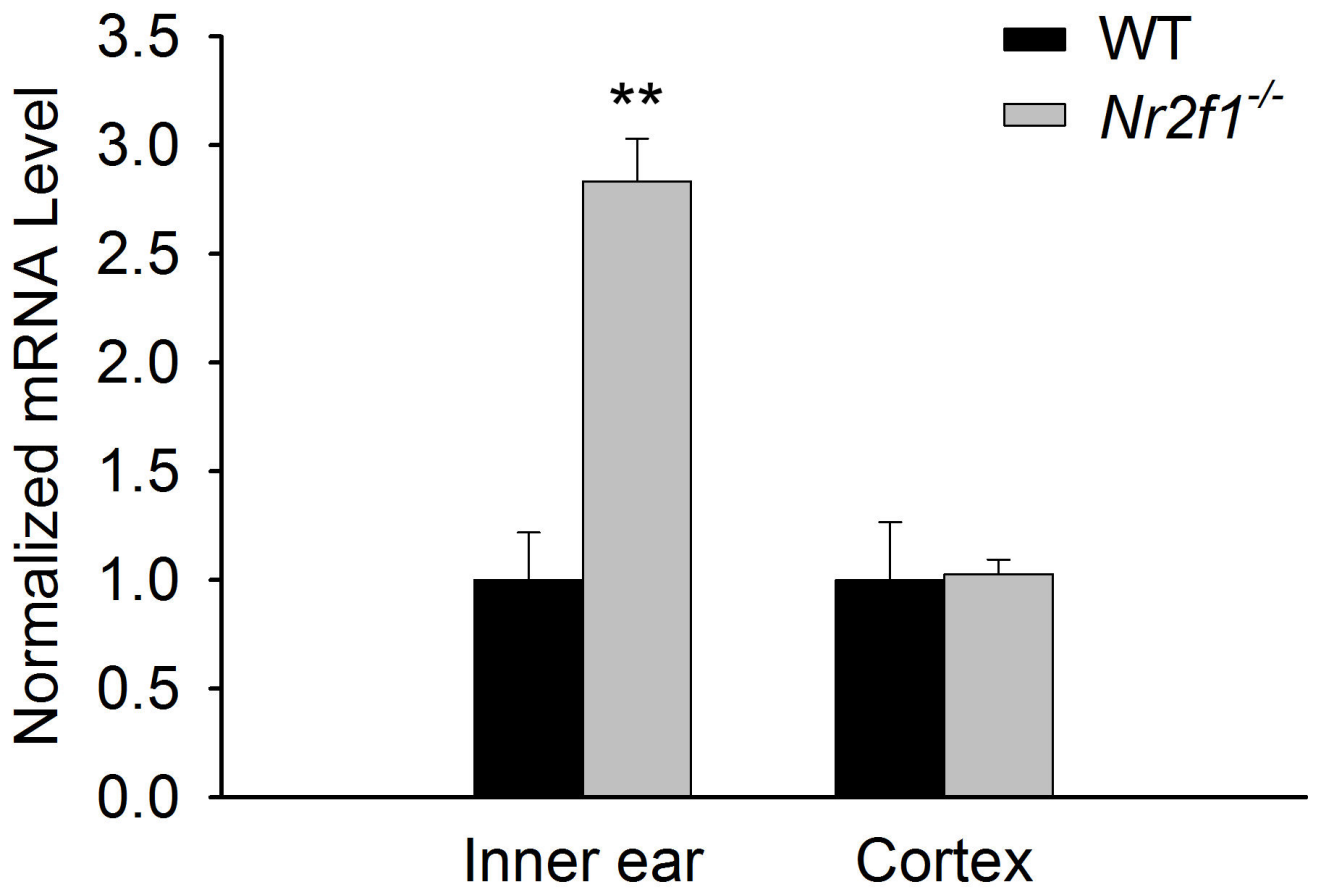
Klf9 is a downstream inner ear target of NR2F1. Klf9 expression is significantly up-regulated in Nr2f1^–/–^ (KO) inner ear but not in the cerebral cortex, as determined by qRT-PCR. N=3. **P<0.01.

**Figure 6:**
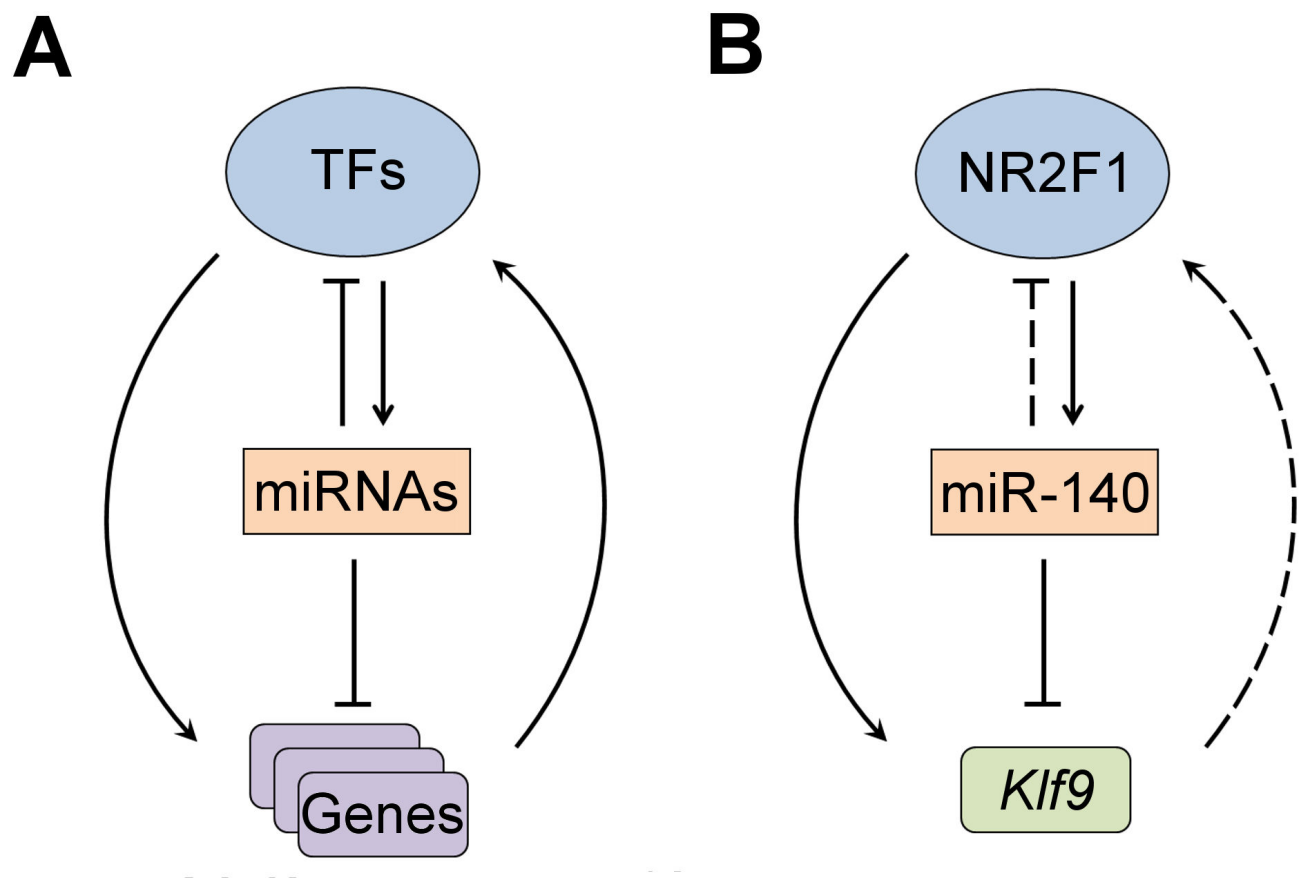
Models of transcription factor-target gene feedback and coregulatory relationships. (A) Generic model showing feedback loops and coregulation between transcription factors (TFs), miRNAs, and genes predicted to be involved in hearing, cancer, and development based on bioinformatic analyses. (B) Model of a validated coregulatory network involving NR2F1, miR-140, and Klf9. Arrow denotes either positive or negative regulation.

Since miR-140 is down-regulated in the absence of NR2F1 only in the inner ear and not the cerebral cortex (Figure **2**) and is a direct target of NR2F1 (Figure **3**), we tested if *Klf9* was regulated in a tissue-dependent manner as well. Indeed, using qRT-PCR, we confirmed that *Klf9* is up-regulated almost 3-fold in the inner ear (P<0.01 vs WT) but was unchanged in the cerebral cortex of *Nr2f1* ^*–/–*^ mice (Figure **5**). Consistent with the luciferase-*Klf9*-3’UTR activity that demonstrated a strong repression of *Klf9* by the miR-140 mimic (Figure **4B**), these data suggest that miR-140 may be a determining factor in regulating *Klf9* expression that is represented in the coregulatory network of NR2F1, miR-140 and *Klf9* (Figure **6**). Together, these experiments provide molecular evidence that *Klf9* is directly coregulated by both miR-140 and NR2F1, further validating the bioinformatically predicted coregulatory network involving NR2F1, miR-140 and *Klf9*. 

## Discussion

Since their discovery approximately 25 years ago [60], there has been great interest in orphan nuclear receptors and their putative ligands. This is based on the knowledge that nuclear receptors and cofactors play crucial roles in many physiological processes during development, reproduction, metabolism and aging [3,61–63]. Orphan nuclear receptors have been discovered to be key components in understanding the regulatory milieu at the genomic scale as they translate cellular, paracrine, neuronal and environmental stimuli into epigenetic and gene expression signals [64–67]. These signals in turn regulate cellular and organismal homeostasis, and dysregulation of this process can lead to diseases including cancer (Tables **S3** & **S6** in File **S1**) [68].

The orphan nuclear receptor NR2F1, located on chromosome 5q14, and its homolog NR2F2, are the most evolutionarily conserved nuclear receptors across all species [69]. NR2F1 is implicated in neurogenesis, cell fate determination, patterning, and regulation of differentiation in the rodent inner ear and cerebral cortex [7–10]. We report here a proof-of-concept novel coregulatory network involving the orphan nuclear receptor NR2F1 and microRNA-140 with experimental validation of the effects these regulators have on each other as well as a mutual target, the *Klf9* gene. This study exemplifies the research approach of using systematic bioinformatic data mining to uncover coregulatory interactions followed by quantitative experimental validations of regulated targets at the transcription, post-transcription and genomic levels. The validation included qRT-PCR, luciferase-3’UTR reporter and ChIP-qRT-PCR assays with tissues from wild type and mutant mice. This approach accomplished several objectives: 1) discovery of a mechanism by which NR2F1 regulates direct target gene expressions; 2) identification of specific miRNAs, such as miR-140, that co-regulate and alter NR2F1 target gene expressions, such as *Klf9*; 3) delineation of a feedback coregulation that occurs between NR2F1 and miR-140; and 4) identification of a tissue-dependent coregulatory network involving NR2F1, miR-140 and *Klf9*.

The *miR-140* gene, located on chromosome 16q22, is most notable for its role in cartilage development and maintenance [70,71]. It also has a suppressive action in liver tumorigenesis [72] and induces pluripotent cells to differentiate into adipocytes [73]. Within the inner ear, miR-140 exhibits differential expression across developmental stages and post-natal maturation [22]. Expression is initially limited to the medial otocyst where cells are proliferating. As development progresses, miR-140 is then expressed in the early sensory epithelium and subsequently in the inner and outer hair cells of the organ of Corti. Lastly, miR-140 is also detected in hair cells of the vestibular apparati by birth [22]. These 3 separate expression patterns suggest miR-140 might have stage-specific roles during inner ear development. Indeed, SOX9 directly activates miR-140 [74] and has a differentially and overlapping expression as SOX2, and is consistent with the miR-140 expression, during cochlear development [75]. This pattern is also highly consistent with that of *Nr2f1* expression during inner ear development [7], supporting the possibility of direct regulation of *miR-140* by NR2F1. Indeed, we found *miR-140* transcripts were significantly down-regulated by 4.5-fold in *Nr2f1* ^*–/–*^ newborn inner ears but remained unchanged in the cerebral cortex (Figure **2**), suggesting NR2F1 acts as a tissue-specific transactivator of *miR-140*. On the other hand, the *Nr2f1* gene itself is a putative target of miR-140 (**Table S2 **in File **S1**), a relationship that may represent a negative feedback loop between NR2F1 and miR-140 as proposed in Figure **6**. Since this finding purports a regulatory feedback loop, we investigated closely the relationship between NR2F1, miR-140, and a mutual target, *Klf9*.

*Klf9*, located on 9q21, acts as a circadian output factor [76], has a role in the regulation of adipogenesis (similar to miR-140) [77], and is up-regulated in a cortisol and differentiation-state-dependent manner. Furthermore, gain- and loss-of-function results in growth arrest and proliferative effects, respectively, in keratinocytes and cancer stem cells [76]. Since all three genes (*Nr2f1*, *miR-140* and *Klf9*) are expressed in the developing inner ear and adult brain (including the cerebral cortex) during neurogenesis, there are other tissues where miR-140, NR2F1, and *Klf9* functionally interact, but the mechanisms of their interactions have not yet been delineated. Indeed, both *KLF9* and NR2F1 have a role in lipid processing [76,77], and miR-140 and *Klf9* are both regulated in the brain by estrogen-receptor modulation [7,22,78]. In the inner ear, estrogen acts to respond to noise-induced damage, and loss of ERβ causes inner ear defects and hearing loss [79–81]. *Klf9* opposes estrogen hormonal action by repressing estrogen receptor (ERα) expression and activity [82–84]. ERα is also known to down-regulate miR-140 in breast cancer stem cells suggesting miR-140 is downstream of estrogen action [83]. The combination of these findings may indicate that the function of mir-140 and *Klf9* is important for estrogen action in the inner ear. 

Further empiric evidence exists of coregulation between miR-140, NR2F1, and *Klf9*. Klf9 is regulated by thyroid hormone and its nuclear receptor TRβ, which are essential for normal development and functional maturation of hearing [85–87]. NR2F1, in turn, is a negative regulator of thyroid hormone receptor binding and thereby suppresses the activation of its target genes (e.g. *Klf9*) [86,88]. Additionally, KLF9 directly suppresses *Notch1* expression and its downstream signaling which promotes differentiation and inhibits glioblastoma neurosphere growth [89]. Indeed, NR2F1 inhibits Notch signaling leading to development and differentiation of extra hair cells and supporting cells in the inner ear [8,20]. Cumulatively, these expression patterns and described actions of NR2F1, *Klf9* and miR-140 are consistent with the co-regulatory network proposed (Figure **6**). Our findings indicate that NR2F1 directly regulates *Klf9* as well as *miR-140*, while miR-140 has a direct inhibitory effect on *Klf9* transcripts. The indirect regulatory effect of NR2F1 by means of miR-140 might have an additive repressive effect than solely by the direct NR2F1targeting of *Klf9*.

In addition to this focused regulation of *Klf9* by a regulatory network involving miR-140 and NR2F1, our bioinformatics search revealed at least 20 other common targets shared by miR-140 and NR2F1, four of which have been validated to have a change of expression in the *Nr2f1* ^*–/–*^ mice [12] (**Table S1 **in File **S1**). *Klf9* is only one of these genes, which according to this model is transactivated at the transcriptional level by NR2F1 but repressed at the post-transcriptional level by miR-140 in order to achieve a balanced expression level. The remaining common targets are also potential miR-140 and NR2F1 co-regulated genes. 

Although we did not study them in depth, our bioinformatics search revealed a number of other possible regulatory networks. There were 107 putative gene targets identified that were shared by NR2F1 and the 11 microRNA that we analyzed. Similar to miR-140, miR-17 and miR-33 target NR2F1 and could therefore be involved in regulatory feedback loops with NR2F1. The majority of the genes (59%) were targeted by multiple miRNA, which may indicate a higher level of control over expression (Figure **1B**). *Pdgfra*, a mutation of which can cause neural tube defects (**Table S3 **in File **S1**) was targeted by NR2F1 and 8 of the miRNAs. Additionally, the miRNAs themselves may act in concert with each other in regulating genes important for ontogenesis and development in general. Using TargetScan 6.2 and microRNA.org to curate the binding sites on the 28 genes targeted by at least 3 of the 11 miRNAs and NR2F1 (Tables S2, S4 & S5 in File **S1**), we discovered two interesting trends. First, different miRNAs (or even the same miRNA in some cases) may bind to overlapping sites on some of the genes. This implicates coordination between the different miRNAs and may allow for a greater regulation of target genes. Also, depending on how many of the regulatory miRNAs are active, this could also allow fine-tuning of the regulation. Second, the same miRNAs may have multiple binding sites. This suggests a greater or more effective regulatory repression and may allow for fine-tuning depending on the accessibility of the different sites under varying conditions [58]. In all, these findings point to the complexity of the interplay between these miRNAs in achieving a balanced regulation of the genes important for development [15].

Furthermore, these coregulatory networks may have clinical significance. The genes targeted by both NR2F1 and the 11 miRNAs that we identified bioinformatically have previously been implicated in hearing loss (*Nr2f1, Crym, Snai2, Gjb2*) [11,90–94] and cancer (*Pdgfrα, Eln, Jun*) [52,95–98], as well as inner ear developmental regulators (*Nr2f1, Notch1, Jag 1, Nptx1, Runx1*) [7,8,22,99], as listed in Tables **S4** & **S5** in File **S1**. *Nr2f1* ^*–/–*^ mice have inner ear developmental defects [7,8,22,99]. Furthermore, miR-140 is selectively expressed in inner ear hair cells during development [22,30] and is demonstrated to regulate targets such as PDGF [100]. It is not surprising that there is a tissue-dependent difference in regulation of *Klf9* in the inner ear compared to the cortex of *Nr2f1* ^*–/–*^ mice given the site-specific regulation of miR-140 and NR2F1. Nor is it unexpected that defects in the cochlear duct, organ of Corti, and hair cells can occur in animals with impaired NR2F1 or miRNA function [7,8,11,16,21,27]. These defects help to underscore the importance of NR2F1 and microRNAs like miR-140, both individually and as coregulatory partners, and give clinical importance to the networked described here.

In conclusion, we have demonstrated that NR2F1 and the 11-selected miRNA may play an important or indispensable role in ontogenesis and development in general. As a proof-of-principle, we experimentally demonstrated the coregulatory mechanisms and feedback loops between NR2F1, miR-140, and *Klf9*. We also uncovered several other potential pathways between NR2F1, miRNAs, and genes important for development and disease. These targets and pathways require further studies and validation in order to advance our understanding of ontogenesis and provide novel strategies for protecting or regenerating hair cells and sensory neurons in patients with hearing loss.

## Supporting Information

File S1 **Supporting Information File containing Tables S1 to S7:**
**Table S1.** Number of predicted miRNAs and NR2F1 inner ear targets.
**Table S2.** Genes targeted by at least 3 of the 11 selected miRNAs.
**Table S3.** Representative list of genes targeted by multiple miRNAs and their associated neurological and other diseases based on the Genetic Association Database.
**Table S4.** List of genes targeted by at least 3 of the 11 select miRNAs and the respective miRNA binding sites in their mRNA 3’ untranslated region based on TargetScan 6.2.
**Table S5.** Genes targeted by at least 3 of 11 selected miRNAs and the respective miRNA binding sites based on miRanda analysis from microRNA.org.
**Table S6.** Functional classification of the genes targeted by miRNAs and NR2F1.
**Table S7.** List of primers for real-time RT-PCR.
(DOCX)
