# Supplementary material for: A Coregulatory Network of NR2F1 and microRNA-140
Source: PLoS One. 2013 Dec 3;8(12):e83358. doi: 10.1371/journal.pone.0083358 (PMC3857795; doi:10.1371/journal.pone.0083358)
Supplement: File S1 — Supporting Information File containing Tables S1 to S7: Table S1. Number of predicted miRNAs and NR2F1 inner ear targets. Table S2. Genes targeted by at least 3 of the 11 selected miRNAs. Table S3. Representative list of genes targeted by multiple miRNAs and their associated neurological and other diseases based on the Genetic Association Database. Table S4. List of genes targeted by at least 3 of the 11 select miRNAs and the respective miRNA binding sites in their mRNA 3’ untranslated region based on TargetScan 6.2. Table S5. Genes targeted by at least 3 of 11 selected miRNAs and the respective miRNA binding sites based on miRanda analysis from microRNA.org. Table S6. Functional classification of the genes targeted by miRNAs and NR2F1. Table S7. List of primers for real-time RT-PCR. (DOCX) [file pone.0083358.s001.docx]

| **Table S1**. Number of predicted miRNAs and NR2F1 inner ear targets. | | | | |
| --- | --- | --- | --- | --- |
| miRNA | miRNA gene targets | Unique miRNA  gene targets | NR2F1 and miRNA co-targeted genes | Co-targeted and validated gene expression changes in NR2F1 knockout [12] |
| mmu-miR-17 | 3024 | 672 | 17 | 4 |
| mmu-miR-33 | 2324 | 549 | 20 | 3 |
| mmu-miR-96 | 3508 | 965 | 29 | 4 |
| mmu-miR-140 | 3576 | 1177 | 20 | 4 |
| mmu-miR-181b | 3048 | 629 | 25 | 0 |
| mmu-miR-183 | 2247 | 500 | 17 | 0 |
| mmu-miR-191 | 1183 | 325 | 8 | 0 |
| mmu-miR-194 | 3290 | 862 | 22 | 2 |
| mmu-miR-199b | 2335 | 570 | 18 | 3 |
| mmu-miR-341 | 850 | 317 | 7 | 2 |
| mmu-miR-1192 | 3537 | 635 | 31 | 6 |
| Total  gene targets | 14639 | 7201 | 107 | 16 |

|  | | **Table S2.** Genes targeted by at least 3 of the 11 selected miRNAs. | | | | | | | | | | | | | | |  |
| --- | --- | --- | --- | --- | --- | --- | --- | --- | --- | --- | --- | --- | --- | --- | --- | --- | --- |
| miR  Target | 17 | | 33 | 96 | 140 | 181b | 183 | 191 | 194 | 199b | 341 | 1192 | No. hits | RMA | Genotype  p-value | dChip | Genotype  p-value |
| *Pdgfra* | X | | X | X | X | X |  |  | X | X |  | X | 8 | 0.95 | 0.0055 | 0.97 | 0.0134 |
| *Ddx3x* |  | | X | X | X | X | X |  |  |  |  | X | 6 | 1.32 | 0.0076 | 1.12 | 0.0431 |
| *Dr1* |  | |  |  |  | X |  | X | X | X |  | X | 5 | 0.87 | 0.0024 | 0.89 | 0.0250 |
| *Sfrs2* | X | | X |  |  |  | X |  | X | X |  |  | 5 | 1.18 | 0.0093 | 1.18 | 0.0082 |
| *Ahnak* | X | | X | X |  | X |  |  |  |  |  |  | 4 | 0.85 | 0.0029 | 0.85 | 0.0367 |
| *Ampd3* | X | | X | X |  |  |  |  |  |  |  | X | 4 | 0.86 | 0.0080 | 0.84 | 0.0009 |
| *Cldn1* | X | |  | X |  |  | X |  | X |  |  |  | 4 | 1.05 | 0.0069 | 0.99 | 0.0002 |
| *Eln* |  | |  |  | X | X |  |  | X | X |  |  | 4 | 0.75 | 0.0007 | 0.84 | 0.0019 |
| *Foxk2* |  | |  | X |  | X | X | X |  |  |  | X | 4 | 1.10 | 0.0463 | 1.24 | 0.0046 |
| *Ldlr* | X | |  | X |  |  |  |  |  | X |  | X | 4 | 1.19 | 0.0075 | 1.20 | 0.0023 |
| *Ran* |  | |  |  |  | X | X |  | X |  |  | X | 4 | 1.18 | 0.0013 | 1.13 | 0.0140 |
| *Sat1* |  | | X |  |  | X |  | X |  | X |  |  | 4 | 0.86 | 0.0244 | 0.78 | 0.0024 |
| *Nr2f1* | X | | X |  | X |  |  |  |  |  |  |  | 3 | 10.34 | 0.0000 | 11.15 | 0.0000 |
| *Acox1* | X | |  |  | X | X |  |  |  |  |  |  | 3 | 0.90 | 0.0312 | 0.91 | 0.0070 |
| *Cd83* |  | |  | X |  | X |  |  | X |  |  |  | 3 | 1.05 | 0.0118 | 1.08 | 0.0035 |
| *Ddit4* |  | |  |  |  | X |  |  |  | X |  | X | 3 | 0.62 | 0.0013 | 0.62 | 0.0010 |
| *Elavl1* |  | |  | X |  |  |  | X |  |  |  | X | 3 | 1.08 | 0.0122 | 1.10 | 0.0079 |
| *Glul* |  | |  | X |  |  |  |  | X |  |  | X | 3 | 0.60 | 0.0088 | 0.70 | 0.0031 |
| *Hmgcs1* |  | |  | X |  |  |  |  | X |  |  | X | 3 | 1.33 | 0.0011 | 1.39 | 0.0010 |
| *Klf9* | X | |  |  | X |  |  |  |  |  |  | X | 3 | 0.73 | 0.0001 | 0.82 | 0.0059 |
| *Man2a1* |  | |  | X | X |  |  |  |  |  |  | X | 3 | 0.87 | 0.0093 | 0.86 | 0.0074 |
| *Nbr1* |  | |  |  |  | X |  | X | X |  |  |  | 3 | 1.15 | 0.0117 | 1.12 | 0.0085 |
| *Nefh* |  | | X |  |  |  | X |  |  |  |  | X | 3 | 1.11 | 0.0326 | 1.16 | 0.2620 |
| *Pros1* |  | |  |  |  | X |  |  |  | X |  | X | 3 | 0.88 | 0.0177 | 0.87 | 0.0066 |
| *Psen1* |  | |  | X |  |  | X |  |  | X |  |  | 3 | 1.19 | 0.0158 | 1.36 | 0.0012 |
| *Ptgis* |  | |  |  | X |  |  |  |  |  | X | X | 3 | 0.83 | 0.0047 | 0.80 | 0.0235 |
| *Stim1* |  | |  | X |  |  |  |  |  | X |  | X | 3 | 0.90 | 0.0125 | 0.90 | 0.0044 |
| *Tbx15* |  | |  | X |  |  | X |  |  |  |  | X | 3 | 0.90 | 0.0054 | 0.97 | 0.0434 |

The RMA (Robust Multichip Average) and dChip (DNA-Chip Analyzer) expression fold-changes WT/KO and respective genotype p-values as calculated from microarray data performed to compare WT versus NR2F1 knockout mouse inner ear tissues as generated by Montemayor *et al.*[12].

| **Table S3.** Representative list of genes targeted by multiple miRNAs and their associated neurological and other diseases based on the Genetic Association Database. A complete list is found here: [NR2F1-miR coregulator targets](http://geneticassociationdb.nih.gov/cgi-bin/bulkview.cgi?geneidtype=gene&genes=Pdgfra%20Ddx3x%20Dr1%20Sfrs2%20Ahnak%20Ampd3%20Cldn1%20Eln%20Foxk2%20Ldlr%20Ran%20Sat1%20Acox1%20Cd83%20Ddit4%20Elavl1%20Glul%20Hmgcs1%20Klf9%20Man2a1%20Nbr1%20Nefl%20Nr2f1%20Pros1%20Psen1%20Ptgis%20Stim1%20Tbx15) | |
| --- | --- |
| Targeted Gene | Associated neurological disease(s) |
| Platelet-derived Growth Factor Receptor A (Pdgfra) | Neural tube defects, Bipolar Disorder, Coronary Artery Disease, Cancers |
| Low Density Lipoprotein Receptor (Ldlr) | Cerebral infarct, Alzheimer’s disease, Atherosclerosis, Obesity |
| Spermidine / spermine N1-acetyltransferase 1 (Sat1) | Depression, Ovarian Cancer |
| Glutamine Synthetase (Glul) | Schizophrenia, Weight Gain |
| Neurofilament Light Polypeptide (Nefl) | Parkinson’s disease, Charcot-Marie-Tooth neuropathy, Alcoholism, Cardiovascular Disease |
| Nuclear Receptor subfamily 2, group F, member 1 (Nr2f1) | Hypertension, Preclampsia |
| Presenilin 1 (Psen1) | Alzheimer’s disease, Frontotemporal dementia |
| Prostaglandin I2 (prostacyclin) synthase (Ptgis) | Cerebral infarction, Hypertension, Myocardial Infarction, Cancers |
| Stromal interaction molecule 1 (Stim1) | Alzheimer’s disease, Cardiovascular Disease |

| **Table S4.** List of genes targeted by at least 3 of the 11 select miRNAs and the respective miRNA binding sites in their mRNA 3’ untranslated region based on TargetScan 6.2. | | | | | | | | | | |
| --- | --- | --- | --- | --- | --- | --- | --- | --- | --- | --- |
| miR**  Target | 17 | 33 | 96 | 140 | 181b | 183 | 191 | 194 | 199b | 1192 |
| *Pdgfra* | 62-68  2757-2763 | 72-78  2065-2072 | 2672-2678 | 59-65 | 2764-2770  2837-2843 |  |  |  | 664-670 | 2206-2212* |
| *Ddx3x* |  | 729-735 | 24-30 | 1300-1306 | 1897-1903  1904-1910  2200-2206  0362-2369 | 887-893 |  |  | 1886-1892 | 294-301*  1494-1500*  2151-2157* |
| *Dr1* |  |  | 1367-1373 |  | 1575-1581 |  |  | 285-291  1104-1110 | 115-121 | 596-602* |
| *Srsf2* | 957-964 | 1930-1936* |  |  |  | 1171-1177 |  |  | 105-111* | 1270-1276 |
| *Ahnak* | 764-771 | 101-107* |  |  | 788-794 |  |  |  |  | 393-399 |
| *Ampd3* | 148-154 | 534-540* | 303-309* | 1322-1328 |  | 343-349 |  |  | 756-762 | 1037-1043* |
| *Cldn1* | 349-355*  760-766* |  | 1138-1144 |  |  | 481-487* |  | 2424-2430 |  |  |
| *Eln* | 393-399  801-807 |  |  | 695-701 | 58-64 |  |  | 532-538* | 597-603 |  |
| *Foxk2* | 2811-2817 | 459-465  1124-1130 | 40-47 | 35-41  89-95  1139-1145 | 1791-1797 | 704-710* | 664-670* | 641-647 | 482-488 | 825-831 |
| *Ldlr* | 815-821  1153-1159  1629-1635  2163-2169 |  | 514-520* | 2434-2440 | 2462-2468 |  |  |  | 322-329* | 2151-2157 |
| *Ran* |  |  |  |  | 152-162 |  |  |  |  | 289-296 |
| *Sat1* |  |  |  |  |  |  |  |  | 297-303 |  |
| *Acox1* | 1736-1742  2298-2304 | 3554-3560 |  | 431-437* | 1228-1234* |  |  | 3800-3806 | 1818-1824 | 2368-2374 |
| *Cd83* |  |  |  |  | 972-979* |  |  | 55-62 | 60-67 |  |
| *Ddit4* |  |  |  | 94-100 | 545-552 | 714-720 |  |  | 507-514 | 286-292*  464-470*  471-477*  836-843* |
| *Elavl1* |  |  | 4628-4634 |  |  | 4212-4218 | 4027-4033  4071-4077 | 3459-3466 | 4268-4274 | 4247-42534247-4253 |
| *Glul* |  |  | 701-707* | 264-270 |  | 1543-1555 |  | 970-976*  1229-1235* | 899-905  1620-1627 | 1262-1272 |
| *Hmgcs1* |  |  | 214-220 | 418-424  471-477  492-498 |  |  |  | 462-468*  821-827* | 600-606 | 821-827  1452-1458 |
| *Klf9* | 790-796 |  |  | 531-537  1416-1422  1457-1464 | 1118-1124 |  |  |  | 391-397 | 518-524  1174-1180 |
| *Man2a1* |  |  |  |  |  |  |  |  |  | 34-40* |
| *Nbr1* | 189-195 |  |  |  | 394-400* |  | 1304-1310* | 131-138*  674-681* |  |  |
| *Nefl* |  | 861-868 |  | 443-449 |  | 1826-1833 |  | 1219-1225 |  | 1308-1314* |
| *Nr2f1* | 90-96 | 164-170 |  | 149-157*  384-194*  453-461* |  |  |  |  |  |  |
| *Pros1* |  |  | 839-845 |  | 624-630*  837-843* |  |  |  |  | 124-130* |
| *Psen1* | 1111-1117 |  |  | 3280-3287 | 3369-3375 | 955-961* |  |  | 2531-2537 |  |
| *Ptgis* | 1276-1282 |  | 3579-3585 |  | 1991-1997 |  |  | 2503-2509  3669-3676 | 639-645  976-982  3714-3720 | 485-491* |
| *Stim1* |  |  | 691-697* | 1406-1412 |  | 1231-1237 |  |  | 832-838* | 518-524 |
| *Tbx15* |  | 1442-1448 | 1643-1649 |  |  | 682-688* |  |  |  | 108-114*  121-127*  831-837* |

*Indicates the binding location was present in TargetScan version 6.0 but not version 6.2.

**miR-341 was not included in Table S4 because no binding sites were found in the TargetScan search.

| **Table S5.** Genes targeted by at least 3 of 11 selected miRNAs and the respective miRNA binding sites based on miRanda analysis from microRNA.org. | | | | | | | | | | |
| --- | --- | --- | --- | --- | --- | --- | --- | --- | --- | --- |
| miR**  Target | 17 | 33 | 96 | 140 | 181b | 183 | 191 | 194 | 199b | 1192 |
| *Pdgfra* | 492-511*  2327-2349  2871-2892# | 58-77^  2025-2045~ | 2677-2698  2783-2805 | 45-64^  405-425  488-509*  1669-1690  2175-2196& | 1078-1100  2875-2899# |  |  |  | 651-672  1516-1538  2008-2029~ | 878-900  2196-2220&  2518-2543  2818-2839  3023-3047 |
| *Ddx3x* |  | 230-250 | 944-966 | 178-199^ | 775-795*  1067-1089  1224-1246 |  |  |  | 196-217^  760-784*  995-1016 | 372-393  1027-1048  1108-1129 |
| *Dr1* |  |  |  |  | 1460-1482 | 650-670 |  | 237-258  583-604* |  | 581-602*  1640-1661 |
| *Sfrs2* |  | 763-783  858-878 |  |  |  | 171-190^ |  | 183-204^ |  | 175-196^  267-288*  286-309*  445-466  935-956 |
| *Ahnak* | 778-801 | 83-105 |  |  | 802-824*  819-841* |  |  |  |  |  |
| *Ampd3* | 124-146  662-684* | 521-541 | 287-309 |  |  |  |  |  |  | 668-689*  1023-1044 |
| *Cldn1* | 328-355  563-586*  743-767 |  | 2188-2211 |  |  | 464-487  1401-1422 |  | 578-599*  1590-1611  1861-1882 |  |  |
| *Eln* |  |  |  | 683-704 | 41-64* |  |  | 518-539 | 28-49* |  |
| *Foxk2* |  |  | 23-44  2133-2154  2212-2234* |  | 1762-1784  2201-2223*  2588-2610  2632-2654 | 715-741 | 679-701 |  |  | 445-466  581-602  855-873 |
| *Ldlr* | 782-804 |  | 19-41  581-603 |  |  |  |  |  | 203-224  386-409 | 977-998*  993-1014*  1009-1130 |
| *Ran* |  |  |  |  | 132-155  1352-1374 | 383-404 |  | 268-290*  868-888  1406-1428 | 946-967 | 267-288*  432-455  1127-1149 |
| *Sat1* |  | 6-25 |  |  |  |  |  |  | 328-349 |  |
| *Acox1* | 406-424* |  |  | 416-437* |  |  |  |  |  |  |
| *Cd83* |  |  |  |  | 755-777  969-989 |  |  | 710-731  1002-1023 |  |  |
| *Ddit4* |  |  |  |  | 544-565 |  |  |  | 505-526 | 462-482  841-861 |
| *Elavl1* |  |  | 2888-2913  3850-3872  4535-4557 |  |  |  |  |  |  | 86-107  421-442  1155-1177  1190-1211  1229-1250  1258-1278  1676-1697  2668-2689  3812-3833  4085-4106  4154-4178 |
| *Glul* |  |  | 685-708 |  |  |  |  | 960-981  1219-1240 |  | 105-126  1122-1143 |
| *Hmgcs1* |  |  | 322-345  1460-1482 |  |  |  |  | 447-468  805-827 |  | 889-911 |
| *Klf9* | 776-800  1651-1673^ |  |  | 471-492*  1416-1437  1460-1480  1650-1671^ |  |  |  |  |  | 260-283  456-479*  1018-1039 |
| *Man2a1* |  |  | 1060-1082 | 1698-1719 |  |  |  |  |  | 13-34  105-126  1663-1684  2148-2171  2237-2258 |
| *Nbr1* |  |  |  |  | 378-401  794-816 |  |  | 117-138  660-681 |  |  |
| *Nefl* |  |  |  |  |  |  |  |  |  |  |
| *Nr2f1* |  | 150-170 |  |  |  |  |  |  | 369-394 |  |
| *Pros1* |  |  |  |  | 612-637  829-850 |  |  |  |  | 115-136  245-267 |
| *Psen1* |  |  | 1050-1072 |  |  | 370-391  942-963 |  |  | 154-173 |  |
| *Ptgis* |  |  |  |  |  |  |  |  |  |  |
| *Stim1* |  |  | 675-697 |  |  |  |  |  | 818-839 | 326-348  498-520 |
| *Tbx15* |  |  | 1577-1601 |  |  | 560-582  690-713 |  |  |  | 94-115*  112-133*  844-962 |

*^&#~ Symbols indicate binding regions for different miRNA which overlap within the respective gene.

**miR-341 was not included in Table S5 because no binding sites were found in the microRNA.org search.

**Table S6.** Functional classification of the genes targeted by miRNAs and NR2F1.

| Cluster*  (Enrichment Score) | Target Genes | Gene Ontology (GO)  Main cellular function(s) |
| --- | --- | --- |
| 1 (1.45) | \| **Crabp1****, **Fabp7**, Rbp1 \| \| --- \| | Cytosolic fatty-acid binding, transport, retinol-binding, cellular hormone metabolic process, forebrain development |
| 2 (1.26) | \| Eif4b, Elavl1, Rbm8a, Sfrs2, Zrsr1 \| \| --- \| | RNA recognition, RNA-binding, mRNA metabolic process, nuclear speck, spliceosome, post-translational regulation |
| 3 (1.13) | \| Cyp51, Hmgcs1, Hsd17b11 \| \| --- \| | Steroid/lipid/cholesterol biosynthesis, |
| 4 (1.07) | \| Mgp, Pros1, Tcn2 \| \| --- \| | Cation transport, vitamin hemostasis, would healing |
| 5 (0.69) | \| Hist1h2ao, H2afy, Satb1 \| \| --- \| | Chromatin assembly/organization, nucleosome core, |
| 6 (0.51) | \| Arg1, Chordc1, Ctbp1, Dr1, Foxk2, **Klf9**, Nbr1, **Nr2f1**, Prrx2, Rasa4, Relb, Satb1, Snai2, Sqstm1, Tbx15, Trip4, Vps25, **Zbtb16**, Zrsr1, 2410022L05Rik \| \| --- \| | Transcription regulation, metal/zinc-binding, regulation of RNA metabolic processes, transcription repressors, embryonic skeletal system development and morphogenesis, alpha-beta T cell differentiation |
| 7 (0.44) | \| Dlk1, Dpep1, Mmp14 \| \| --- \| | Skeletal system development, metalloproteases, tube development and morphogenesis, regulation of cell-matrix adhesion |
| 8 ((0.36) | \| Casq1, Dlk1, Man2a1, Pros1, **Stim1**, \| \| --- \| | Calcium ion binding/transport, endomembrane system, response to wounding |
| 9 (0.06) | \| Ak1, Ampd3, Cd83, Cldn1, Ddx3x, Dhx40, Dlk1, Gjb2, **Glul**, Ltc4s, Ms4a6b, Myadm, Ndufb6, Pdgfra, Slc25a1, Tek, Timm23, Tmem106a, Tyro3, Vps4a \| \| --- \| | ATP binding, cell adhesion, carboxylic acid biosynthesis, mitochondrion inner membrane, sex differentiation, skeletal development |

*The lowest stringency in the Gene Functional Classification analysis was used to discover 9 clusters containing 66 genes using DAVID. Note: 41 genes excluded from analysis.

**Genes in Bold type were previously validated to be changed in NR2F1 knockout by qRT-PCR [12].

**Table S7.** List of primers for real-time RT-PCR.

| Gene | Forward Sequence 5’-3’ | Reverse Sequence 5’-3’ |
| --- | --- | --- |
| Klf9 | TGGAGAGTCCCGATGAGGATA | GAGGCGTGTTTCCCCTTCG |
| CycloA | TAAAGCATACGGGTCCTGGC | TGTTGGGTCCAGCATTTGC |
| miR-140 promoter (1) | GGCAGAGAGTCCTAGCAGTG | AGTCAGATGACAGCCCCACA |
| miR-140 promoter (2) | AGCACTGTGTTACCTTCACCC | CCTTCCCACTGCTAGGACTC |
| ChIP control | GTTGTGGAACCATGTGCGAGT | CTTTTCAGCATGAATTGGGTCTG |
